# Be Well Baytown: Whole-Community Cancer Prevention Initiative Based on Multi-Sector Capacity and Partnership Building

**DOI:** 10.1177/10732748251347584

**Published:** 2025-06-02

**Authors:** Margaret Raber, Katherine Oestman, Lori Rumfield, Rosemary Coffman, Nikki Rincon, Mayra Aquino, Brad Love, Michael T. Walsh, Ruth Rechis

**Affiliations:** 1 Department of Health Disparities, 4002 University of Texas MD Anderson Cancer Center, Houston, TX, USA; 2 4002 University of Texas MD Anderson Cancer Center, Cancer Prevention Platform, Houston, TX, USA; 3 127032 Goose Creek Consolidated Independent School District, Baytown, TX, USA; 4 1570 Lee College, Baytown, TX, USA; 5 Hearts and Hands of Baytown, Baytown, TX, USA; 6 University of Texas, Moody School of Communication, Center for Health Communication, Austin, TX, USA

**Keywords:** community health, modifiable risk factors for cancer, multi-sector coalition, evidence-based interventions

## Abstract

The objective of this paper is to describe 7 years of implementation of a multi-sector, whole community cancer prevention program and share lessons learned in multi-sector capacity and partnership building. Be Well Baytown is led by the University of Texas MD Anderson Comprehensive cancer center and is centered around a community coalition (Steering Committee) based in Baytown, Texas. The program aimed to implement evidence-based interventions (EBIs) to address modifiable risk factors for cancer through local Collaborating Organizations. EBI implementation, investments in capacity building (ie, technical assistance, program evaluation, sustainability planning, etc.), Steering Committee member partnerships, and overall reach of the initiative were assessed. Eight Collaborating Organizations across 6 sectors received more than 3500 capacity building hours to plan and deliver EBIs in the community between 2018 and 2024. The initiative sustained reach over the first 7 years of implementation with 93% of the city population reached in 2024. Partnerships between organizations increased over time with Steering Committee members (n = 17) reporting an average of 14.1 partners in 2024. Whole-community interventions require initial and sustained buy-in across multiple sectors. The experience of the program over 7 years demonstrates the potential for whole-community interventions that prioritize cross-sectoral collaboration and local capacity building.

## Introduction

Recent changes related to the designations of Comprehensive Cancer Centers (CCCs) have created new opportunities to build synergy between tertiary cancer centers and public health efforts. In 2016, the National Cancer Institute began implementing community outreach and engagement (COE) requirements for centers that receive national designation. The goal of COE programming is to enhance cancer prevention and control throughout the areas CCC institutions serve with an emphasis on reducing health disparities, enhancing access for underserved communities and populations, and working with local stakeholders to optimize uptake of cancer preventive behaviors. This guidance is aligned with place-based approaches to health promotion. Place-based approaches are tailored to local environments and communities and aim to address local environmental conditions and systems that influence health and health behavior by working alongside residents and key community partners and enhancing existing community assets.

The promise of multi-sector interventions to promote cancer preventive behaviors, coupled with NCI recommendations to CCCs to engage with local partners, offer strong rationale for aligning institutional COE efforts with large-scale place-based initiatives. As part of The University of Texas MD Anderson Cancer Center’s mission to end cancer, Be Well Communities™ was launched as the institution’s place-based strategy for comprehensive cancer prevention and control, working with communities to reduce modifiable risk factors for cancer.^1^ Detailed elsewhere, the Be Well Communities model places MD Anderson as the leading, backbone organization in health promotion efforts, with MD Anderson staff engaging community organizations and local government as well as residents to plan, implement, evaluate, and sustain interventions to reduce cancer risk, building from existing health promotion frameworks.^1,2^ To date, three Be Well Communities have been initiated in Pasadena,^3^ Baytown,^1,2,4,5^ and the Acres Homes^6^ neighborhood in Houston, Texas.

Be Well™ Baytown was launched at the end of 2016 with planning occurring in 2016-2017 and implementation starting in 2018. Baytown, Texas is the third largest city in Harris County, just east of Houston. It is a majority-minority city of over 83 000 residents, with 47% identifying as Hispanic, and 18% as African American. Nearly one third (31.3%) of Baytown residents are uninsured. Baytown adults report levels of tobacco use 65% higher than the national average of 11.5%. Over one-third (38.4%) of adults have obesity, and 30.3% do not engage in physical activity outside of work.^7^

Be Well Baytown and all Be Well Communities engage a diverse array of community partners across multiple sectors to facilitate the creation of a community coalition,^8^ a key recommendation for health promotion by the World Health Organization.^9^ Community coalitions have been shown to improve the effectiveness and sustainability of health promotion programs.^8,10-12^ Coalition building requires the development of partnerships with and investment in existing community assets.^13^ Multi-sector collaboration for health promotion allows for connectivity between system environments (socioeconomic, policy, environmental, healthcare) that influence health. This connectivity can then be leveraged into interventions that influence multiple levels of the social-ecological model (ie, individual, interpersonal, community, societal).

There are several existing frameworks for effectively engaging diverse sectors in health promotion that inform the Be Well Communities approach. The Robert Wood Johnson Foundation’s Framework for Aligning Systems for Health^14^ highlights key aspects to successfully engaging the healthcare, public health, and social services sectors toward common community health goals. In this and other models, shared vision and community engagement and partnerships are central common elements, further supported by governance, leadership, financing, interventions, continuous learning, and data/measurement.^15,16^ Operationalizing the Aligning Systems for Health and related frameworks is informed by theories of behavior change, notably Michie’s COM-B (Capability + Opportunity + Motivation = Behavior) model^17^ which posits that capability, opportunity, and motivation are the key influencers of human behavior and thus key targets for behavior change intervention. Springer et al.’s Health Interweaving Model^18^ offers a pathway to linking COM-B to intersectoral community coalition frameworks by describing how health promotion elements can be integrated into different environments (informational, physical, policy, sociocultural, etc.) within community sectors to impact these influencers. Community coalition and behavior change theory inform how multi-sector interventions create opportunities for individuals to engage in sustained healthy lifestyle behaviors. Interweaving health promotion components into organizational structures then helps sustain programming after initial investments end.

The objective of this paper is to describe the first 7 years of implementation of a multi-sector, whole community cancer prevention program, Be Well Baytown, and share lessons learned in multi-sector capacity and partnership building.

## Methods

### Be Well Baytown Structure

The development of the Be Well Baytown Steering Committee, subsequent Community Action Plan (CAP) and role of MD Anderson as the backbone organization are described elsewhere.^1^ Briefly, the Be Well Baytown Steering Committee is a community health coalition consisting of representatives from organizations that plan and guide implementation of evidence-based interventions (EBIs) in the area alongside residents of the community. Organization representatives who were invited to serve on the Steering Committee were identified through information-gathering interviews with established community leaders (ie, the school district superintendent). Additionally, those initial organizations were asked to recommend other key stakeholders operating in the community (ie, FQHC leadership) to join the Steering Committee. Sectors represented on the Be Well Communities Steering Committee include education, healthcare, community organizations and local government (detailed in Table 1). The Steering Committee is tasked with promoting and guiding the initiative in the community, reviewing and prioritizing EBI implementation, and supporting and monitoring programs over the multi-year initiative.

**Table 1.** Sectors Participating in Be Well Baytown, Specific Organizations, programs^EBIs^ Implemented, and Select Assessment Metrics.

Sector	Organization	Focus Area: Program	Metrics
K-12 education	Goose creek consolidated independent school district	Healthy eating/Active living^a^	Healthy eating/Active living
		CATCH	Proportion of GCCISD students/School campuses implementing CATCH
		Safe walking trails	Sun safety
		Sun safety^b^	# Shade structures installed
		Shade structure installation	Proportion of GCCISD students receiving sun safety education
		Sun safety education	Tobacco-free living
		Tobacco-free living^c^	# of students participating in ASPIRE
		ASPIRE	# of students participating in this is quitting
		This is quitting	Preventive care
		Preventive care^d^	# of students receiving HPV vaccination
		HPV vaccination clinic	
Local government	City of baytown parks & recreation department	Active living^e^	Active living
		Pop up park events	# of pop up park events
		Baytown moves campaign	Sun safety
		Sun safety^f^	# Shade structures installed
		Shade structure installation	Proportion of eligible parks and recreation staff receiving sun protective clothing
		Sun protective clothing distribution	Proportion of eligible parks and recreation staff attending sun safety education
		Sun safety education	
Public health	Harris county public health	Healthy eating/Active living^g^	Healthy eating/Active living
		Project OLE Texas for early childcare and education centers	# of early childcare centers participating in project OLE Texas
		Safe routes to schools and parks	# Students impacted by OLE
		Sun safety^h^	# Students participating in walk to school events
		Mobile sunscreen units	Sun safety
		Sun safety education	# Mobile sunscreen units
			Proportion of eligible project OLE Texas staff attending sun safety education
Higher education: Community college	Lee college	Sun safety^i^	Sun safety
		Shade structure installation	# Shade structures installed
		Campus sunscreen dispensers	# of sunscreen dispensers installed
		Sun safety media campaign	Proportion of students reached with sun safety media campaign
		Tobacco free living^j^	Tobacco free living
		Tobacco free living media campaign	Proportion of students reached with tobacco media campaign
		Tobacco cessation resources for students	# of tobacco specialists provided
Social services	Hearts and hands of baytown & Houston Food bank	Healthy eating^k^	Healthy eating
		Healthy food distribution program	Pounds of food distributed
			# of mobile food distribution events
Healthcare	Chambers health	Preventive care^l^	Preventive care
		Patient navigation	# of cancer screenings completed
		Comprehensive screening programs	

Evidence based interventions:

^a^Multicomponent School-based Obesity Prevention Interventions, Active recess, community-based social support for physical activity programs, physically active classrooms, places for physical activity, school-based physical education enhancements, screen time interventions for children, walking school buses.

^b^Primary and middle school based interventions for skin cancer, multicomponent community-wide interventions for skin cancer.

^c^Comprehensive tobacco control programs, mass media campaigns against tobacco use.

^d^Schools and organized childcare centers vaccination programs.

^e^Community fitness programs, multicomponent obesity prevention interventions.

^f^Interventions in outdoor occupational settings to prevent skin cancer, interventions in outdoor recreational settings to prevent skin cancer.

^g^Nutrition and physical activity interventions in preschool and child care, Complete Streets and Streetscape Design Initiatives, Safe routes to school.

^h^Child care center based interventions to prevent skin cancer, multicomponent community-wide interventions to prevent skin cancer.

^i^Interventions in outdoor occupational settings to prevent skin cancer, Multicomponent community-wide interventions to prevent skin cancer.

^j^Comprehensive tobacco control programs, mass media campaigns against tobacco use, smoke-free policies for indoor areas.

^k^Healthy food initiatives in food pantries.

^l^Group education for clients for breast cancer screening, Multicomponent interventions for cancer screening, patient navigators, provider reminder and recall systems, reducing client out-of-pocket costs for cancer screening, reducing structural barriers for cancer screening.

Focus priorities of Be Well Baytown include healthy eating, active living, sun safety, tobacco-free living, and preventive care (vaccination, screening). The planning process for development of the mutually agreed-upon CAP are explained in detail elsewhere.^6^ Collaborating Organizations are funded Steering Committee organizations that are responsible for delivering the EBIs in the CAP and working collaboratively with other members of the Steering Committee to optimize program implementation and plan for long term sustainability. The Be Well Communities team has a track record of successful localization of efforts after initial funding ends.^19^ Initial funding for the 10-year Be Well Baytown initiative was made possible through generous philanthropic donations to MD Anderson of $10 million dollars.

### Be Well Communities Capacity and Partnership Building

The Be Well Communities team supports capacity building for individual Collaborating Organizations. Collaborating Organizations enter a service-agreement with MD Anderson for expenses related to EBI implementation and evaluation (such as staff salaries, program fees, equipment, infrastructure, data collection costs). Funding decisions consider clear, detailed sustainability plans and declared intents of organizations. Collaborating Organizations work with the Be Well Communities team to detail implementation, evaluation/metrics, and sustainability plans through an iterative process that aims to optimize organization impact both throughout the project period and after the initial funding ends.

Be Well Communities staff facilitate partnership and cross-sector collaboration between organizations. This includes connecting Collaborating Organizations to resources and other organizations to support program implementation success, sharing best practices for doing work in this space, and troubleshooting issues. This curated partnership building between Collaborating Organizations aims to build an aligned implementation community within a local ecosystem that makes partnership and requests for support easy and expected.

### Partnership and Capacity Building Metrics

The collective work and functioning of the Steering Committee and Collaborating Organizations is measured by the number of organizations represented on the Steering Committee each year and results from the annual Stakeholder Survey. The survey is completed by 1-2 representatives from each Steering Committee organization and examines attitudes about the initiative, functioning of the coalition, and the number of partnerships developed among members. Capacity building metrics include the number of EBIs implemented as part of the initiative and the number of person-hours staff dedicated by the Be Well Communities team.

### Collaborating Organizations, EBIs, and Metrics

Be Well Baytown Collaborating Organizations represent K-12 education, city government, public health, higher education, and social services. The specific organizations representing each sector, the EBIs they were tasked with implementing, and their reach and implementation metrics are shown in Table 1. These metrics are collected through quarterly and annual reports from Collaborating Organizations. Overall reach is calculated as the number of individuals exposed to Be Well Baytown EBIs and events, divided by the total population of Baytown. Results for each quarter were summarized by year from inception (2018) to 7 years (2024). Metrics related to behavior change have been reported elsewhere.^20^

## Results

### Steering Committee Function, Partnership and Capacity

**Figure 1.**
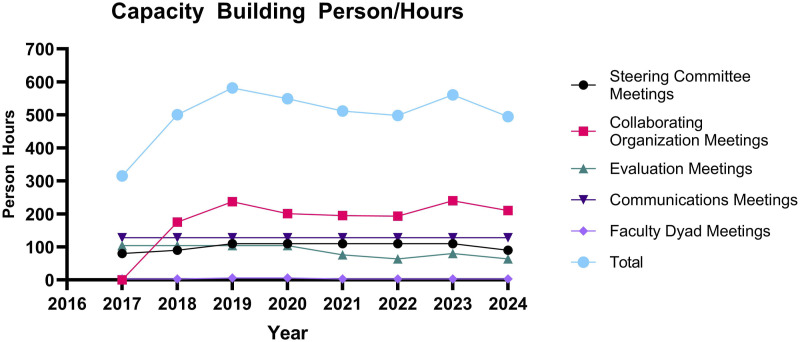
Capacity building hours completed by MD Anderson staff team total (blue) and by sub-category. 2016 is considered a planning year, therefore personnel hours were not calculated.

Between 2016/2017, the planning year, and 2024, the Be Well Communities team dedicated more than 4000 capacity building person hours to the project. Capacity building activities included Steering Committee meetings (810 h), Collaborating Organization meetings (1452 h), evaluation meetings (700 h), communications meetings (1024 h), and faculty dyad meetings (27 h). Total time commitment varied over the course of the project (Figure 1). Collaborating Organization meetings commenced in 2017, but person hours by other capacity building activities remained relatively stable. Collaborating Organization meetings, wherein the Be Well Communities team worked directly with partners across sectors to implement EBIs (Table 1), utilized the most person hours. Faculty dyad meetings, in which specialists in public health and population science were engaged as advisors to Be Well Communities team, utilized the least person hours.

**Figure 2.**
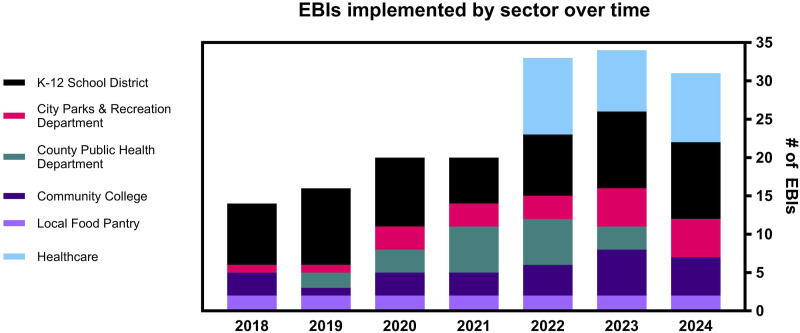
Evidence Based Interventions (EBIs) implemented in Be Well Baytown by sector over 7 years. Chambers Health (light blue), was funded as a collaborating organization in 2022.

The trajectory of Be Well Baytown once implementation began (2018), including the number of EBIs by sector, is shown in Figure 2. Total EBIs continued to increase over the first 6 years of the project, reaching an apex of 34 cancer prevention EBIs in 2023 as both individual sectors and the community coalition expanded capacity.

Supporting Collaborating Organizations, 20 additional organizations were represented on the Steering Committee in 2017. Membership changed slightly over the course of the program and varied between 22-25 members. Response rates to the Stakeholder Survey also varied from a low in 2020 of 60% (n = 13 completed surveys) to a high of 86% in 2022 and 2023 (n = 19 completed surveys). Average response rates across seven years of the program was 77%. Sector representative characteristics and key findings from the Stakeholder Survey are shown in Table 2. Most respondents identified as representing a Community Organization (such as local food pantry), Education (K-12 and community college), Government (city parks and recreation, public health department) or Healthcare sectors (such as hospitals, local clinics). The proportion of representatives changed over time, with healthcare representative participation dipping in 2020 to 8%. Stakeholder survey respondents widely agreed that the Steering Committee included members from relevant sectors and that members participated actively during meetings. Overall, respondents reported a positive view of Steering Committee functioning, including the role of Steering Committee members in supporting Collaborating Organizations in carrying out EBIs.

**Table 2.** Stakeholder Survey Results 2018-2024 (Select Items).

Year (n)	2018 (19)	2019 (16)	2020 (13)	2021 (18)	2022 (19)	2023 (19)	2024 (17)
Respondent sector n(%)							
Community organization	9 (47.4)	7 (43.8)	4 (33.3)	8 (44.4)	7 (36.8)	7 (36.8)	6 (42.9)
Education	6 (31.6)	4 (25.0)	4 (33.3)	6 (33.3)	6 (31.6)	6 (31.6)	5 (35.7)
County/local government	4 (21.1)	3 (18.8)	3 (25.0)	4 (22.2)	2 (10.5)	4 (21.1)	2 (14.3)
Healthcare	4 (21.1)	2 (12.5)	1 (8.3)	4 (22.2)	5 (26.3)	3 (15.8)	2 (14.3)
Other	3 (15.8)	1 (6.3)	0 (0.0)	1 (5.6)	1 (5.3)	0 (0.0)	1 (7.1)
Partnership items							
Is your organization partnering with other organizations? n(%)							
Yes	9 (75.0)	11 (100.0)	8 (100.0)	9 (69.2)	10 (83.3)	—	—
No	2 (16.7)	0 (0.0)	0 (0.0)	0 (0.0)	1 (8.3)	—	—
Not sure	1 (8.3)	0 (0.0)	0 (0.0)	4 (30.8)	1 (8.3)	—	—
How many different organizations is your organization partnering with to carry out the program activities as part of be well baytown? n(%)							
1-2	0 (0.0)	2 (18.2)	0 (0.0)	5 (55.6)	0 (0.0)	2 (20.0)	0 (0.0)
3-5	3 (33.3)	3 (27.3)	3 (60.0)	0 (0.0)	4 (40.0)	3 (30.0)	2 (25.0)
6+	4 (44.4)	4 (36.4)^*a*^	2 (40.0)	2 (22.2)	2 (20.0)	5 (50.0)	5 (62.5)
Don’t know/not sure	2 (22.2)	2 (18.2)	0 (0.0)	2 (22.2)	4 (40.0)	2 (16.7)	1 (12.5)
Agreement (1 strongly disagree – 5 strongly agree) with the following statements: Mean (SD)							
We have developed new partnerships in the community through this initiative.	4.9 (0.3)	4.7 (0.5)	4.9 (0.3)	4.8 (0.7)	4.8 (0.4)	4.6 (0.7)	4.7 (0.7)
We have strengthened existing partnerships in the community through this initiative.	4.9 (0.3)	4.8 (0.4)	4.9 (0.3)	4.7 (0.7)	4.5 (0.9)	4.6 (0.7)	5.0 (0.0)
We have funding (other than Be well baytown funding) with one or more partners to expand work related to this initiative.	3.6 (0.9)	3.9 (0.5)	3.8 (1.1)	3.8 (1.3)	4.1 (1.3)	3.5 (1.3)	4.2 (0.8)
Steering committee/Coalition items							
Agreement (1 strongly disagree – 5 strongly agree) with the following statements: Mean (SD)							
The steering committee includes members from multiple relevant sectors.	4.8 (0.5)	4.8 (0.8)	5.0 (0.0)	4.9 (0.3)	—	—	—
All steering committee members participate actively in initiative meetings.	4.3 (0.6)	4.2 (0.7)	4.3 (0.6)	4.3 (0.7)	—	—	—
Steering committee members help to coordinate the activities of collaborating organizations to achieve this initiative’s goals.	4.3 (1.1)	4.7 (0.5)	4.6 (0.7)	4.7 (0.6)	4.4 (0.8)	4.4 (1.1)	4.5 (0.7)
Steering committee members support collaborating organizations to carry out program activities effectively.	4.5 (0.7)	4.8 (0.6)	4.7 (0.5)	4.8 (0.5)	4.6 (0.5)	4.6 (0.8)	4.6 (0.6)
The steering committee is working well together to assess progress of this initiative.	4.7 (0.6)	4.8 (0.6)	4.7 (0.6)	4.6 (0.6)	4.5 (0.8)	4.5 (0.6)	4.6 (0.7)

**Figure 3.**
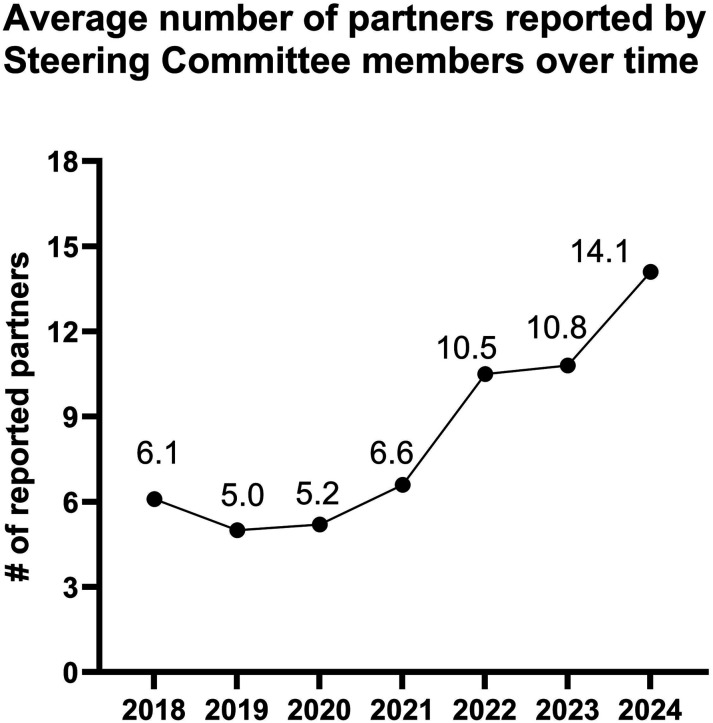
Average number of partners reported by Be Well Baytown Steering Committee members by year over 7 years.

The majority of Stakeholder Survey respondents reported partnering with other organizations. Coalition partnership reached a high in 2019 and 2020 with 100% of respondents noting inter-organizational partners. Partnership overall was at its lowest during the COVID-19 pandemic in 2021, with 69.2% of members reporting partnerships. The average number of organizational partners reported by Steering Committee members increased over time, with an average of 14.1 partners reported by organizations in 2024 (Figure 3).

Overall, Steering Committee members agreed that the initiative led to the creation of new partnerships and strengthened existing partnerships. The availability of funding for partnerships changed over time, with the greatest percentage of respondents “somewhat” or “strongly” agreeing that their organization had funding with one or more partners to expand work related to the initiative in 2019 (82%) and 2022 (80%).

### Overview of Impact by Sector/Collaborating Organization

The partnerships and collaborations detailed above enabled implementation of EBIs within and across sectors. Estimated program impact and reach by sector/collaborating organization from 2018-2024 is detailed below. Community reach during the year expanded in tandem with EBI implementation with 35% of the total Baytown population reached in 2018 up to 93% (78 583 individuals) in 2024.

#### K-12 Education

Goose Creek Consolidated Independent School District (GCCISD) implemented a multi-component school-based intervention for obesity prevention including district-wide implementation of the Coordinated Approach To Child Health (CATCH) program in elementary and middle schools, 23 of 29 campuses by the 2018-2019 school year. Sun safety EBIs implemented by the district included infrastructure investments (14 sunshade structures) and sun safety education implemented by all 29 schools across grade levels. GCCISD junior and high schools implemented evidence-based tobacco-free living initiatives and a total of 3059 students participated in tobacco cessation programs by 2022 (data not reported in 2023/24). Regarding preventive cancer care, 57 GCCISD free vaccination clinics hosted over the course of the project provided all recommended vaccinations, including the HPV vaccine to 1154 adolescents, in partnership with UTHealth, a Steering Committee organization. Implementation of EBIs in GCCISD was supported by UTHealth and the YMCA of Baytown. Approximately 30 000 students and staff were reached by EBIs annually.

#### Higher Education

Lee College is a local, Hispanic-Serving Institution located in Baytown. Lee College implemented sun safety interventions including the installation of 10 sunshade structures, 16 on campus sunscreen dispensers, student education, and a sun safety media campaign. Lee College also prioritized tobacco-free living interventions including a tobacco-free living media campaign and tobacco cessation resources for students, maintaining two Certified Tobacco Treatment Specialists and integrating tobacco control curricula into five classes. Approximately 8500 students and staff were reached by EBIs annually.

#### Local Government

The City of Baytown Parks and Recreation Department (PARD) implemented EBIs related to active living and sun safety. Baytown PARD implemented a media campaign “Baytown Moves” and hosted 22 “Pop Up Park” events with over 4200 people from 2017-2024 to encourage physical activity and park utilization among residents. Baytown PARD also installed 7 sunshades, 23 non-permanent umbrellas, distributed protective clothing to 100% of employees working outdoors and provided sun safety education to employees. To further encourage park utilization, PARD made improvements to existing structures, including revitalizing two park entrances.

#### Public Health

Harris County Public Health (HCPH) implemented the Outdoor Learning Environment (OLE! Texas) program which includes active living and healthy eating curriculum and infrastructure improvements at four early childcare and education centers throughout Baytown and reached ∼250 young children by 2022. HCPH implemented a safe routes to schools and parks program in collaboration with GCCISD and PARD, which aimed to improve walkability by identifying and remediating potential pedestrian hazards through adding additional street lighting, crosswalks, and traffic signage, and hosting events to encourage walking to school or local parks. By 2022, 1001 students had participated in walk-to-school events. HCPH implemented sun safety education for 36 childcare center staff participating in OLE! Texas; and added sun protection elements to its fleet of mobile public health units by providing sunscreen and sun safety education materials during community outreach and events.

#### Social Services

Hearts and Hands of Baytown is a faith-based organization that offers free food distributions to community members experiencing food insecurity. Hearts and Hands partners with the Houston Food Bank and the United Way of Greater Baytown Area and Chambers County (UWGBACC) to implement healthy food distribution programs at their pantry and expanded mobile distribution events. Prior to Be Well Baytown, Hearts and Hands hosted 10 events per year at one pantry. Between 2017 and 2024, over 9 million pounds of healthy food were distributed in the area, including distributions at 424 mobile food distribution events. Four active food pantries hosted over 80 events each year. Work was supported by UWGBACC by ensuring connections between social-sector agencies and increasing use of appropriate services through the free 211 helpline.

#### Healthcare

Chambers Health is a federally qualified health center serving the greater Baytown area. Chambers Health participated in the MD Anderson’s Certified Tobacco Treatment Training Program (CTTTP), which provides a 5-day training for individuals interested in becoming credentialed as Tobacco Treatment Specialists. Chambers Health also implemented a patient navigation program to integrate referrals to cancer screenings. From 2022-2024, 5812 cancer screenings were provided (mammograms, colon tests, pap tests). Health center annual data of quality reporting indicated the patient navigation program resulted in an increased number of screenings completed as well as increased compliance with screening each year of the program.

## Discussion

Be Well Baytown supported the implementation of EBIs through nine collaborating organizations and 20 additional supportive organizations- forming a multi-sector coalition for health. Education, government, public health, and social services organizations implemented increasing numbers of EBIs in the community and demonstrated sustained reach and impact over the first 7 years of implementation. Reflection on the barriers and facilitators to effective partnership and capacity building informs ongoing sustainability planning as well as future COE investments.

### Lessons Learned

Be Well Baytown serves as a model for other CCCs aiming to prevent cancer through place-based efforts, engaging community coalitions and investing time and resources to build organizational capacity and facilitate collaboration across community sectors. The initiative supports EBIs at multiple levels, supporting community members in a way that is aligned to the nuances of the socioecological model. Baytown residents gain opportunities for healthier living through changes to the environmental infrastructure such as parks, organizational changes such as healthy eating and exercise initiatives in schools, and enhanced preventive care for individuals through increased cancer screening access. Capacity of local organizations to offer health promotion activities is supported through MD Anderson’s technical and financial assistance as the backbone organization, and motivation to integrate sustainable cancer prevention activities into organizational structure is enhanced through the Steering Committee’s shared central agenda of improving community health. Be Well Baytown centers on the activation of existing community assets (including existing parks and recreation infrastructure, clinics, schools, non-profit organizations and local government) to form a place-based ecosystem of health promotion implementers,^21^ operationalizing the Aligning Systems for Health Framework in a diverse community setting. The model further relies on being fully present and engaged in the community setting as an active member of the community.

A unique aspect of Be Well Baytown’s approach was the high person-hours dedicated to working alongside Collaborating Organizations as they planned, implemented, and evaluated EBIs aligned with the Steering Committee’s CAP, established in the planning year (2016). A key aspect of the Be Well Communities approach was setting clear expectations with Collaborating Organizations early in the program implementation. This included detailing the types of support and services that the team could offer organizations, including evaluation assistance. One expectation was required, at least bi-weekly meetings with all Collaborating Organizations, which offered an opportunity for ensuring successful implementation of the EBIs, formalizing metrics and evaluation procedures for accountability tracking, and streamlining the facilitation of partnerships as representatives from other organizations could join the standing meetings as opportunities for collaboration arose. These capacity building activities empowered organizations to offer health promotion programming in a way that resonated with their community; and their local expertise was critical to the successful implementation of multi-level interventions throughout Baytown.^21^

To accommodate the high standards of data-driven program implementation, Collaborating Organizations needed dedicated staff. One strategy to enhance organizational capacity was direct funding for staff positions. Organizations that requested support for new positions were required to outline sustainability plans for after the service agreement ends. Funding staff through Be Well Baytown helped create internal champions for the program across Collaborating Organizations, minimizing potential barriers to organizational changes needed to accommodate programming.

There are issues when supporting new staff for organization partners including potential mismatch between community/program needs and the person/people recruited by the organization to fill those roles. Additionally, turnover can negatively impact progress as trained staff move on to other jobs before the service agreement is complete. Unanticipated changes to staff, as well as other factors, can also impact service agreement budgets and lead to excesses or dearth of funds. The Be Well Communities team needed to become adept at helping organizations change budgetary needs and cope with carry-over funds.

The broader Be Well Baytown Steering Committee received positive feedback from members, including high agreement that members help coordinate and support the Collaborating Organizations. The creation of a multi-sector ecosystem for health is further reflected in the increasing numbers of partners members reported working with over the course of the project. As partnerships grew, the capacity of organizations followed suit, with numbers of EBIs implemented in the community growing annually. Notably, not all sectors implemented equal numbers of EBIs, and sectors expanded and contracted efforts at different times across the project years. Partially, this may be due to the partnership building aspect of Be Well Communities, which encourages Collaborating Organizations to find similarities in their work plans to effectively distribute programming. One example of this includes HCPH’s safe routes to school program. While GCCISD had a distinct interest in transportation for students, the public health department had the staff and skills to assess the local environment, while the City of Baytown had the authority to implement road improvements.

There are strengths to this work, including the analysis of Stakeholder Surveys across 7 years of intervention, which allowed for comparison over years. Additionally, a variety of metrics were utilized by the Collaborating Organizations and centralized with the Be Well Communities team. There are limitations to this report, such as variability in response to the Stakeholder Survey year over year, and the exclusion of other outcome metrics that are beyond the scope of this article. Another limitation is the complexity of measuring reach in a multi-level, whole-community intervention setting, wherein the utilization of some efforts, such as infrastructure improvements, are not easily captured. Additionally, Be Well Baytown occurred in a time period that included multiple disasters in the region that had prolonged effects including Hurricane Harvey (2016), COVID-19 (2020) and the Texas winter storm (2021), which impacted programming efforts. Despite these limitations, this work offers insight into the scope and structure of a multi-sector whole-community health promotion/cancer prevention effort undertaken through a CCC.

## Conclusion

Be Well Communities provides a real-world example of the operationalization of a multi-sectoral approach to individual and community-health promotion. Whole-community interventions require initial and sustained buy-in across multiple sectors. The experience of Be Well Baytown over 7 years highlights the potential for transformative change through collaborative planning with community partners, capacity building for existing community assets, and the creation of partnership networks to build an ecosystem of health promotion.

## Data Availability

Data is available to researchers upon written request to the authors.*
